# Efficacy and safety of liraglutide in patients with type 2 diabetes mellitus and severe obstructive sleep apnea

**DOI:** 10.1007/s11325-022-02768-y

**Published:** 2022-12-21

**Authors:** Wenlong Jiang, Weiguo Li, Jing Cheng, Wen Li, Fangzhou Cheng

**Affiliations:** 1 Department of Cardiovascular, Shenzhen Yantian District People’s Hospital, Shenzhen, China; 2 Department of Cardiovascular, Longyan First Hospital Affiliated to Fujian Medical University, Longyan, Fujian China

**Keywords:** Liraglutide, 2 diabetes mellitus, Apnea–hypopnea index, Obstructive sleep apnea, Body mass index

## Abstract

**Objectives:**

To observe the efficacy and side effects of liraglutide in the treatment of type 2 diabetes mellitus (T2DM) patients with severe obstructive sleep apnea (OSA).

**Methods:**

The study conducted in an outpatient setting was a two-center, prospective randomized controlled study. T2DM patients with severe OSA were randomized to the control group (continuous positive airway pressure [CPAP] and drug treatment without liraglutide) or the liraglutide group (CPAP and drug treatment including liraglutide). Demographic and clinical characteristics, sleep-disordered breathing indices, cardiac function indices, and side effects were evaluated and compared between the two groups before and after 3 months.

**Results:**

Of 90 patients, 45 were randomized to the intervention arm (with liraglutide) and 45 to the control arm (without liraglutide). One patient in the liraglutide group dropped out of the study on day 8 after enrollment due to obvious gastrointestinal symptoms. No significant differences were found between the two groups in baseline demographics, clinical characteristics, cardiac function indicators, or sleep disorder respiratory indices (*P* > 0.05). After 3 months, the body mass index (BMI), apnea hypopnea index (AHI), and mean systolic blood pressure in the liraglutide treatment group were significantly lower than those in the control group (*P* < 0.05). The minimum oxygen saturation was significantly higher in the liraglutide group compared with that in the control group after 3 months of follow-up (*P* < 0.05). No difference was found between the two groups in the summary of side effects (*P* > 0.05).

**Conclusions:**

Liraglutide combined with CPAP can effectively reduce BMI, lower mean systolic blood pressure, and improve AHI scores and hypoxia in T2DM patients with severe OSA. Liraglutide did not increase side effects.

## Introduction

Severe obstructive sleep apnea (OSA) is often associated with insulin resistance, which increases the risk of type 2 diabetes mellitus (T2DM) in patients with OSA. Untreated OSA can affect glycemic control, increase the incidence of cardiovascular and cerebrovascular diseases in patients with OSA patients and T2DM, and affect the prognosis of OSA with T2DM [1]. The latest research suggests that diabetics who are treated with insulin are at higher risk for OSA. The prevalence of OSA in patients with T2DM with obesity has been reported to be 26 to 83% [2–4]. There is a bidirectional relationship between OSA and T2DM [5]. Intensive lifestyle interventions, diet control, weight loss, and continuous positive airway pressure (CPAP) are all current treatment options for patients with severe OSA [6]. Currently considered effective treatment outcomes include diet, exercise, and/or CPAP [7]. However, patients have difficulty adhering to these treatment routes long-term, especially weight loss interventions [8]. CPAP may also lead to weight gain, further exacerbating metabolic complications [9, 10]. Therefore, the optimal treatment for the patients with T2DM and severe OSA is to provide glucose control and weight loss interventions in addition to CPAP. Obesity and insulin resistance are often seen in patients with T2DM and severe OSA, which is the theoretical basis for providing liraglutide treatment for these patients. We investigated the effects and safety of liraglutide regarding sleep apnea indicators, weight control, cardiac function, and glycolipid metabolism in patients with T2DM and severe OSA. 

## Methods

This study was registered in Science and Technology Bureau of Shenzhen Yantian District (No. 20180329). The Ethics Committee of Shenzhen Yantian People’s Hospital approved the study in accordance with the principles of the Declaration of Helsinki. It was a randomized, controlled, nonblinded clinical trial in patients with T2DM and severe OSA. The clinical trial was conducted in Shenzhen Yantian District People’s Hospital and Longyan First Hospital Affiliated to Fujian Medical University from July 2019 to September 2021. All patients visited the clinic once a month for a follow-up period of up to 3 months.

### Patient enrollment

From July 2019 to September 2021, patients with T2DM and severe OSA (AHI > 15) were recruited from clinics at Shenzhen Yantian People’s Hospital and Longyan First Hospital Affiliated to Fujian Medical University. All patients who participated in this study provided informed consent.

Inclusion criteria were:

1. Age 18–75 years;
2. Clinical diagnosis of T2DM;
3. Severe OSA with apnea-hypopnea index (AHI) > 15 by polysomnography;
4. No current use of DPP-IV inhibitors or GLP-1RA treatment;
5. Current use of CPAP treatment.

Exclusion criteria were:

1. Pregnant, breastfeeding, or planning to become pregnant soon;
2. Contraindication for DPP-IV inhibitors or GLP1-RAs;
3. Heart failure class III–IV;
4. Chronic kidney disease stage 4 and stage 5;
5. History of pancreatitis;
6. Infections, trauma, hyperthyroidism, hypothyroidism, or malignant tumors;
7. Reluctance to follow up or participation in other clinical trials simultaneously.

The patients were followed up in the outpatient clinic once per month and prescribed medication. One patient in the liraglutide group refused to continue the study because of nausea and vomiting on day 8. All follow-up and data collection were completed in the remaining 89 patients.

### Study protocol and measures (Fig. 1)

**Fig. 1:**
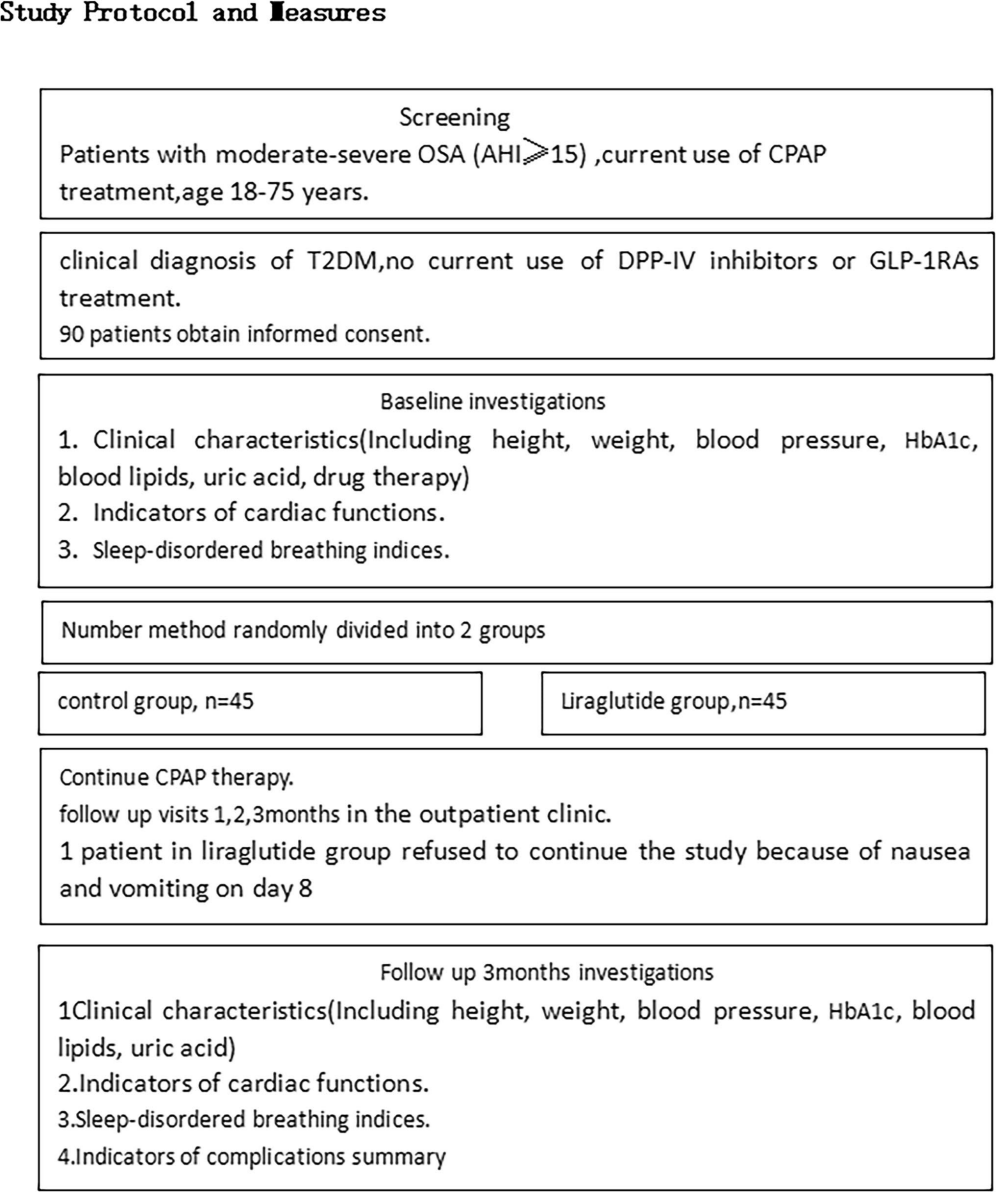
Study protocol and measures

#### Control group (no intervention)

The control group was not given a placebo. If conventional hypoglycemic drugs (excluding liraglutide) were given for poor blood glucose control, the type or dosage of the drugs could be adjusted these adjustments were recorded. All patients were treated with CPAP for OSA.

#### Liraglutide group

Liraglutide was injected subcutaneously once daily into the abdomen, thigh, or upper arm. It was commenced at a starting dose of 0.6 mg once per day. The dose was increased to 1.2 mg/day after 1 week and 1.8 mg/day after the second week. Those who did not tolerate the increased dose continued to maintain the original dose of 0.6 mg/day or 1.2 mg/day. If the drug was tolerated, the patient was required to maintain the maximum tolerated dose. All adjustments occurred under the guidance of the research team. Patients continued CPAP treatment during this period.

### Clinical characteristics

Measurements recorded at baseline and follow-up included body mass index (BMI), blood pressure, uric acid levels, blood lipid levels, HbA1c levels, and medication use (hypertension, diabetes, and hyperlipidemia medications).

### Detection of cardiac function indicators

The left ventricular ejection fraction (LVEF) and left ventricular end-diastolic diameter (LVEDd) were measured by echocardiography at baseline and after 3 months of follow-up. M-type echocardiography was used to measure LVEF.

### Sleep-disordered breathing indices

At baseline and after 3 months of follow-up, polyrespiratory sleep monitoring was performed, and AHI and minimum nocturnal oxygen saturation were recorded.

### Summary of the indicators of side effects

Side effects including gastrointestinal complaints, hypoglycemia, pancreatitis, and dermatitis during treatment with liraglutide were recorded at each follow-up.

### Follow-up

Follow-up was completed in the outpatient clinic once per month and continued for 3 months. 

### Statistical analysis

SPSS 19.0 software (SPSS, Chicago, IL) was used for statistical analysis. Continuous variables with a normal distribution are expressed as the mean ± standard deviation (SD), while continuous variables with a non-normal distribution are expressed as the median and quartile difference. Categorical variables are expressed in absolute values and percentages. For comparison between the two groups, Student’s *t* test was used for normally distributed values, and the Mann‒Whitney *U* test was used for non-normally distributed values. Categorical variables were compared using the chi-square test or Fisher’s exact test. Intragroup (before and after 3 months of treatment) comparisons, including comparisons of the liraglutide group and control group, were performed by paired sample *t* tests (when continuous variables followed a normal distribution) and Wilcoxon tests (when continuous variables followed a non-normal distribution). *P* < 0.05 (2-tailed) was considered statistically significant.

## Results

### Baseline clinical data

Demographic and clinical data are summarized in Table 1. There were no significant differences in demographic and clinical characteristics at baseline between the control group and the liraglutide group (*P* > 0.05) (Table 1). Of the 90 patients in this study, one patient in the liraglutide group dropped out because of significant gastrointestinal discomfort giving a lost to follow-up rate of 1.1%.

**Table 1:** Baseline of demographic, clinical characteristics

Variables	Liraglutide group (*n* = 44)	Control group (*n* = 45)	*P* value
Age (years)	55.7 ± 7.4	54.8 ± 5.5	0.554
Male (*n* [%])	34	29	0.245
Smoking (*n* [%])	18	13	0.271
Hypertension (*n* [%])	31	36	0.334
ACE inhibitors (*n* [%])	25	30	0.387
CCBs (*n* [%])	19	25	0.292
β-Blockers (*n* [%])	9	12	0.619
Diuretics (*n* [%])	12	10	0.629
Metformin (*n* [%])	34	36	0.800
Sulfonylurea (*n* [%])	19	22	0.672
Thiazolidinedione (*n* [%])	7	9	0.784
α-Glucosidase inhibitor (*n* [%])	12	15	0.646
SGLT-2 (*n* [%])	6	8	0.772
Insulin (*n* [%])	9	14	0.183
Cholesterol-lowering drugs (*n* [%])	15	19	0.515

*BMI*, body mass index; *ACE*, angiotensin-converting enzyme; *CCBs*, calcium channel blockers; *24hSBP*, 24-h dynamic mean systolic blood pressure; *24 h DBP*, 24-h dynamic mean diastolic blood pressure

### Cardiac functions

The LVEF and LVEDd values at baseline and 3 months after randomization were measured to estimate cardiac function. The LVEF and LVEDd values were not significantly different between the control group and the liraglutide group at baseline and 3 months after randomization (*P* > 0.05) (Table 2).

**Table 2:** Indicators of cardiac functions

Variables	Liraglutide group (*n* = 44)			Control group (*n* = 45)			*P* value between two groups	
	Baseline	After 3 months	*P* value in group	Baseline	After 3 months	*P* value in group	Baseline	After 3 months
LVEF (%)	63.8 ± 5.70	64.4 ± 3.8	0.567	63.5 ± 4.2	62.8 ± 4.7	0.447	0.789	0.097
LVEDd (mm)	48.4 ± 3.47	47.5 ± 3.3	0.137	48.6 ± 3.0	46.2 ± 3.9	0.052	0.73	0.099

*LVEF*, left ventricular ejection fraction; *LVEDd*, left ventricular end-diastolic diameter

### Sleep-disordered breathing indices

At baseline, there was no significant difference in AHI and minimum oxygen saturation between the two groups, but after 3 months of randomization, the liraglutide group had significantly better AHI and minimum oxygen saturation than the control group (*P* < 0.05). The AHI and minimum oxygen saturation values were also significantly better in the liraglutide group after 3 months of treatment than at baseline (*P* < 0.05) (Table 3).

**Table 3:** Sleep-disordered breathing indices

Variables	Liraglutide group (*n* = 44)			Control group (*n* = 45)			*P* value between two groups	
	Baseline	After 3 months	*P* value in group	Baseline	After 3 months	*P* value in group	Baseline	After 3 months
AHI	31.0 ± 7.3	26.1 ± 7.1	0.000	30.1 ± 6.22	31.6 ± 6.9	0.246	0.537	0.000
Minimum oxygen saturation (%)	80.3 ± 5.8	83.4 ± 5.8	0.000	80.4 ± 5.0	80.4 ± 5.9	0.991	0.953	0.017

*AHI*, apnea–hypopnea index

### Indicators of cardiovascular risk

BMI, blood lipids, uric acid, HbA1c, and 24-h ambulatory blood pressure were measured after 3 months of follow-up. The BMI and 24-h mean systolic blood pressure (SBP) values were lower in the liraglutide group than in the control group after the 3-month follow-up (*P* < 0.05). The BMI, CHOL, LDL, HbA1c and 24-h ambulatory blood pressure values were significantly improved in the liraglutide group after 3 months compared with baseline, and the BMI and HbA1c values in the control group were also improved after 3 months compared with baseline (Table 4).

**Table 4:** Indicators of cardiovascular risk

Variables	Liraglutide group (*n* = 44)			Control group (*n* = 45)			*P* value between two groups	
	Baseline	After 3 months	*P* value in group	Baseline	After 3 months	*P* value in group	Baseline	After 3 months
BMI, kg/m [2]	26.5 ± 4.4	24.4 ± 4.5	0.000	27.0 ± 2.4	26.9 ± 2.4	0.000	0.478	0.001
CHOL, mmol/L	5.63 ± 0.63	5.32 ± 0.65	0.000	5.71 ± 0.73	5.54 ± 0.62	0.288	0.586	0.102
LDL, mmol/L	3.78 ± 0.73	3.71 ± 0.73	0.000	3.76 ± 0.53	3.84 ± 0.64	0.503	0.827	0.353
Uric, μmol/L	359.27 ± 88.93	334.52 ± 91.74	0.192	347.47 ± 112.12	351.04 ± 95.85	0.876	0.584	0.409
HbA1c, %	6.64 ± 0.61	6.41 ± 0.61	0.000	6.64 ± 0.50	6.57 ± 0.50	0.000	0.979	0.203
24hSBP, mmHg	130.4 ± 11.9	124.8 ± 12.5	0.000	132.2 ± 13.0	131.0 ± 13.0	0.636	0.509	0.024
24hDBP, mmHg	75.0 ± 8.8	72.5 ± 8.1	0.007	74.4 ± 9.3	74.4 ± 9.6	0.993	0.773	0.305

*BMI*, body mass index; *ACE*, angiotensin-converting enzyme; *CCBs*, calcium channel blockers; *24hSBP*, 24-h dynamic mean systolic blood pressure; *24hDBP*, 24-h dynamic mean diastolic blood pressure

### Summary of the indicators of side effects

No pancreatitis occurred during treatment with liraglutide at any follow-up timepoint. No significant differences in the occurrence of gastrointestinal complaints, hypoglycemia, or dermatitis were found between the two groups (*P* > 0.05) (Table 5).

**Table 5:** Indicators of side effects

Variables	Liraglutide group (*n* = 44)	Control group (*n* = 45)	*P* value
Gastrointestinal complaints, *n*	7	4	0.353
Hypoglycemia, *n*	2	1	0.616
Dermatitis, *n*	2	1	0.616

## Discussion

Liraglutide is a hypoglycemic agent used to treat T2DM. It is a glucagon-like peptide-1 receptor agonist (GLP-1RA) and can also be used in weight loss therapy [11]. A dose of 1.8 mg/day is commonly used to treat diabetes, while a dose of 3.0 mg/day or higher is used for weight control. The highest dose used in this study was 1.8 mg/day. OSA is often associated with T2DM. As a routine treatment for OSA, CPAP has the risk of affecting metabolism, and patients have poor compliance with CPAP treatment. Therapies that benefit both OSA and T2DM patients are important. Weight loss is beneficial for controlling blood sugar levels and improving OSA. Our study suggested that liraglutide, a GLP-RA, is a hypoglycemic agent associated with significant weight loss that improves the severity of OSA in T2DM patients.

Several large studies in patients with diabetes have found that liraglutide improves blood sugar control and leads to significant weight loss [12]. The SCALE diabetes trial involving obese patients (BMI greater than 27) showed that liraglutide given subcutaneously at 3.0 and 1.8 mg resulted in 6% and 5% weight loss, respectively, compared to placebo. In our study, the maximum intervention dose of liraglutide was 1.8 mg once daily. The results showed a 2.0 decrease in BMI in the liraglutide group. These results suggested that liraglutide 1.8 mg once a day has a good effect on weight loss in the Chinese population. The weight loss effect of liraglutide is primarily achieved through appetite suppression mediated by peripheral and central nervous system (CNS) pathways. It can directly stimulate POMC neurons while inhibiting arcuate nucleus neuropeptide y (NPY) and AgRP neurons, reducing hunger and increasing satiety [13]. This feeling of fullness can also have an effect on other areas of the brain, such as the mesolimbic system. These effects lead to a loss of appetite and feelings of nausea. Therefore, liraglutide injections reduce stomach motility and slow the emptying of stomach contents, affecting food intake and leading to weight loss [14, 15]. The latest study showed that 5% weight loss did not affect the risk of cardiovascular events, but 10% weight loss was enough to reduce cardiovascular events [16]. Although BMI decreased significantly, lipid levels, uric acid levels, glycosylated hemoglobin levels, and cardiac function indicators did not improve significantly in our study, which was the result of 3-month follow-up. Longer follow-up studies are needed to confirm whether liraglutide treatment can improve blood lipid, uric acid, glycated hemoglobin levels, and other indicators. However, our study also showed that the 24-h mean SBP in the liraglutide group was significantly lower than that in the control group. This may be due to improvements in AHI and minimum oxygen saturation values at night.

The SCALE sleep apnea study showed weight loss and improved AHI scores in the liraglutide group, which was consistent with the conclusions of this study [17]. In addition, liraglutide improved the minimum blood oxygen saturation in patients with severe OSA and T2DM [18]. There is a strong association between obesity and OSA. Risk factors for OSA include tonsil and adenoid hypertrophy and obesity, which may lead to upper airway stenosis or obstruction [19]. Corrado Pelaia found in their study that BMI was closely related to neck circumference, waist circumference [20], peripheral oxygen saturation, and AHI scores [21]. Additional studies have shown that regular physical activity may reduce the occurrence of severe OSA in an evidence-based way through weight loss [22]. Our study showed significant weight loss in the liraglutide group, which may reduce adipose tissue compression of the upper airway or prevent airway collapse. This improves OSA severity, reduces AHI scores, and increases nighttime minimum oxygen saturation. More studies are needed to determine if liraglutide improves the sleep disorder respiratory index in other ways.

OSA is a risk factor for hypertension, coronary heart disease, arrhythmia, heart failure, stroke, and other cardiovascular and cerebrovascular diseases [23, 24]. The underlying mechanism of the association between OSA and cardiovascular disease is not yet fully understood. The current first-line treatment for OSA is CPAP. However, there are some problems. First, the results from randomized controlled trials suggest that CPAP therapy does not reduce the incidence of cardiovascular disease [25]. Second, there is the concern of long-term adherence to CPAP therapy. This study suggests that liraglutide, as a GLP-RA and a hypoglycemic agent associated with weight loss for the treatment of type 2 diabetes, may improve OSA severity. This study suggests that liraglutide, as a GLP-RA and a hypoglycemic agent associated with weight loss, may improve OSA severity in patients with T2DM. However, whether or not liraglutide improves cardiovascular risk factors in T2DM patients with severe OSA remains unclear. Some studies have shown that liraglutide improves various cardiometabolic parameters in obese and prediabetic patients, reducing the risk of diabetes [26]. Our study showed that after 3 months of liraglutide intervention, the mean SBP in patients with OSA and T2DM was controlled. However, no significant difference was found in the average SBP, blood lipid level, glycosylated hemoglobin level, and uric acid level in the liraglutide group, which may be related to the insufficient liraglutide intervention time and the small sample size of this study.

GLP-1 agonists and analogs have been reported to be well tolerated. Nausea is the most common symptom of discomfort during early liraglutide treatment [27]. Although this side effect is not a serious health concern, it may cause patients to stop liraglutide treatment [28]. Liraglutide is associated with hypoglycemic risk, but severe hypoglycemic episodes were rarely reported in the LEAD study. In our study, there was no difference in the incidence of gastrointestinal discomfort, hypoglycemia, or dermatitis between the two groups.

Although these results are novel, there are limitations to our study that need to be considered. First, despite being a two-center study, the sample size was not large, which limits the ability to generalize the findings. Second, follow-up time was limited and the effects on lipids, blood sugar, and other indicators need to be confirmed by longer term follow-up studies. Third, our findings cannot be generalized to patients with mild OSA because only patients with severe OSA were enrolled.

## Conclusion

Liraglutide is a glucose-lowering drug used in patients with T2DM and for weight loss in obese patients. In our study, liraglutide combined with CPAP improved the severity of OSA, lowered BMI and mean SPB values in patients with T2DM and severe OSA, and did not increase hypoglycemic side effects.

## Data Availability

The datasets used and/or analyzed during the current study are available from the corresponding author upon reasonable request.

## References

[1] Devaraj NK (2020). Knowledge, attitude, and practice regarding obstructive sleep apnea among primary care physicians. Sleep and Breathing.

[2] Coughlin SR, Mawdsley L, Mugarza JA (2004). Obstructive sleep apnoea is independently associated with an increased prevalence of metabolic syndrome. Eur Heart J.

[3] Foster GD, Sanders MH, Millman R (2009). Obstructive sleep apnea among obese patients with type 2 diabetes. Diabetes Care.

[4] West SD, Nicoll DJ, Stradling JR (2006). Prevalence of obstructive sleep apnoea in men with type 2 diabetes. Thorax.

[5] Huang T, Lin BM, Stampfer MJ (2018). A population-based study of the bidirectional association between obstructive sleep apnea and type 2 diabetes in three prospective US cohorts. Diabetes Care.

[6] Giles TL, Lasserson TJ, Smith BH et al (2006) Continuous positive airways pressure for obstructive sleep apnoea in adults. Cochrane Database Syst Rev CD001106 10.1002/14651858.CD001106.pub2 16437429

[7] Foster GD, Borradaile KE, Sanders MH (2009). A randomized study on the effect of weight loss on obstructive sleep apnea among obese patients with type 2 diabetes: the sleep ahead study. Arch Intern Med.

[8] Ashrafian H, le Roux CW, Rowland SP (2012). Metabolic surgery and obstructive sleep apnoea: the protective effects of bariatric procedures. Thorax.

[9] Craig SE, Kohler M, Nicoll D (2012). Continuous positive airway pressure improves sleepiness but not calculated vascular risk in patients with minimally symptomatic obstructive sleep apnoea: the mosaic randomised controlled trial. Thorax.

[10] Drager LF, Brunoni AR, Jenner R (2015). Effects of CPAP on body weight in patients with obstructive sleep apnoea: a meta-analysis of randomised trials. Thorax.

[11] Food and Drug Administration. Prescribing information for Saxenda, 2018 [Internet]. Maryland: Food and Drug Administration; 2018 [cited 2020 May 29]

[12] Buse JB, Nauck M, Forst T (2013). Exenatide once weekly versus liraglutide once daily in patients with type 2 diabetes (DURATION-6): a randomised, open-label study. Lancet.

[13] Secher A, Jelsing J, Baquero AF (2014). The arcuate nucleus mediates GLP-1 receptor agonist liraglutide-dependent weight loss. J Clin Invest.

[14] Dailey MJ, Moran TH (2013). Glucagon-like peptide 1 and appetite. Trends Endocrinol Metab.

[15] Williams DL, Baskin DG, Schwartz MW (2009). Evidence that intestinal glucagon-like peptide-1 plays a physiological role in satiety. Endocrinology.

[16] Wing RR, Lang W, Wadden TA (2011). Benefits of modest weight loss in improving cardiovascular risk factors in overweight and obese individuals with type 2 diabetes. Diabetes Care.

[17] Collier A, Blackman A, Foster G et al (2014) S28 liraglutide 3.0 mg reduces severity of obstructive sleep apnoea and body weight in obese individuals with moderate or severe disease: Scale Sleep Apnoea Trial. BMJ Publishing Group Ltd

[18] Patinkin ZW, Feinn R, Santos M (2017). Metabolic consequences of obstructive sleep apnea in adolescents with obesity: a systematic literature review and meta-analysis. Child Obes.

[19] Khan MA, Mathur K, Barraza G (2020). The relationship of hypertension with obesity and obstructive sleep apnea in adolescents. Pediatr Pulmonol.

[20] Pelaia C, Armentaro G, Miceli S (2021). Association between sleep apnea and valvular heart diseases. Front Med (Lausanne).

[21] Rezaie L, Maazinezhad S, Fogelberg DJ, Khazaie H, Sadeghi-Bahmani D, Brand S (2021). Compared to individuals with mild to moderate obstructive sleep apnea (OSA), individuals with severe OSA had higher BMI and respiratory-disturbance scores. Life (Basel).

[22] Hall KA, Singh M, Mukherjee S, Palmer LJ (2020). Physical activity is associated with reduced prevalence of self-reported obstructive sleep apnea in a large, general population cohort study. J Clin Sleep Med.

[23] Urbanik D, Martynowicz H, Mazur G (2020). Environmental factors as modulators of the relationship between obstructive sleep apnea and lesions in the circulatory system. J Clin Med.

[24] Tietjens JR, Claman D, Kezirian EJ, Yeghiazarians Y (2019). Obstructive sleep apnea in cardiovascular disease: a review of the literature and proposed multidisciplinary clinical management strategy. J Am Heart Assoc.

[25] Labarca G, Dreyse J, Drake L, Jorquera J, Barbe F (2020). Efficacy of continuous positive airway pressure (CPAP) in the prevention of cardiovascular events in patients with obstructive sleep apnea: systematic review and meta-analysis. Sleep Med Rev.

[26] Rezaie L, Phillips D, Khazaie H (2018). Barriers to acceptance and adherence to continuous positive airway pressure therapy in patients with obstructive sleep apnea: a report from Kermanshah province, western Iran. Patient Prefer Adherence.

[27] Pi-Sunyer X, Astrup A, Fujioka K (2015). A randomized, controlled trial of 3.0 mg of liraglutide in weight management. N Engl J Med.

[28] Buse JB, Rosenstock J, Sesti G (2009). Liraglutide once a day versus exenatide twice a day for type 2 diabetes: a 26-week randomised, parallel-group, multinational, open-label trial (LEAD-6). Lancet.

